# A Predictive Based Regression Algorithm for Gene Network Selection

**DOI:** 10.3389/fgene.2016.00097

**Published:** 2016-06-15

**Authors:** Stéphane Guerrier, Nabil Mili, Roberto Molinari, Samuel Orso, Marco Avella-Medina, Yanyuan Ma

**Affiliations:** ^1^Department of Statistics, University of Illinois at Urbana-ChampaignChampaign, IL, USA; ^2^Research Center for Statistics, Geneva School of Economics and Management, University of GenevaGeneva, Switzerland; ^3^Department of Statistics, University of South CarolinaColumbia, SC, USA

**Keywords:** biomarker selection, genomic networks, disease classification, breast cancer, acute leukemia, model averaging

## Abstract

Gene selection has become a common task in most gene expression studies. The objective of such research is often to identify the smallest possible set of genes that can still achieve good predictive performance. To do so, many of the recently proposed classification methods require some form of dimension-reduction of the problem which finally provide a single model as an output and, in most cases, rely on the likelihood function in order to achieve variable selection. We propose a new prediction-based objective function that can be tailored to the requirements of practitioners and can be used to assess and interpret a given problem. Based on cross-validation techniques and the idea of importance sampling, our proposal scans low-dimensional models under the assumption of sparsity and, for each of them, estimates their objective function to assess their predictive power in order to select. Two applications on cancer data sets and a simulation study show that the proposal compares favorably with competing alternatives such as, for example, Elastic Net and Support Vector Machine. Indeed, the proposed method not only selects smaller models for better, or at least comparable, classification errors but also provides a set of selected models instead of a single one, allowing to construct a network of possible models for a target prediction accuracy level.

## 1. Introduction

Gene selection has become a common task in most gene expression studies. The problem of assigning tumors to a known class is an example that is of particular importance and has received considerable attention in the last 10 years. Conventional class prediction methods of leukemia or other cancers are in general based on microscopical examination of stained tissue specimens. However, such methods require highly trained specialists and are subjective (Tibshirani et al., 2002).

To avoid these drawbacks, many automatic selection methods have been proposed recently. The goal of these methods is often to identify the smallest possible set of genes that can still achieve good predictive performance (Díaz-Uriarte and De Andres, 2006), although this is not necessarily the only criterion based on which model (gene) selection is carried out (see for example Leng et al., 2006). However, these methods have the advantage of being objective and have improved the correct classification rate in various cases. Among the different methodologies brought forward in this context we can find those proposed by Tibshirani et al. (2002), Dudoit et al. (2002), Zhu and Hastie (2004), and Zou and Hastie (2005). See also Díaz-Uriarte and De Andres (2006) and the references therein for other approaches.

Nonetheless, many of these methods do not necessarily respond to the needs of practitioners and researchers when they approach the gene selection process. First of all, many of them have to rely on some form of size reduction and often require a subjective input to determine the dimension of the problem. Also, many of these methods often provide a single model as an output whereas genes interact inside biological systems and can be interchangeable in explaining a specific response. The idea of interchangeability of genes in explaining responses appears for instance in Kristensen et al. (2012). These authors use the PARADIGM algorithm of Vaske et al. (2010) to combine mRNA expression and DNA copy number in order to construct clusters of patients that provide the best predictive value. The resulting clusters can be seen as being characterized by different significantly expressed genes and we can refer to their interactive structure as *paradigmatic* networks.

Another issue of most existing gene selection methods is their reliance on the likelihood function, or a penalized version of it, as a means to develop a selection criterion. However, the likelihood function may not necessarily be the quantity that users are interested in as they may want to target some other kind of loss function such as, for example, the classification error. Of course, maximizing the likelihood function is not typically the same as minimizing a particular loss function. Moreover, adapting these methods to handle missing or contaminated data is not straightforward. This has limited the applicability and reliability of these methods in many practical cases.

To eliminate the limitations of the gene selection procedures described above, this paper proposes an objective function for out-of-sample predictions that can be tailored to the requirements of practitioners and researchers. This is achieved by enabling them to select a criterion according to which they would like to assess and/or interpret a given problem. However, the optimization of such a criterion function is typically not an easy task since the function can be discontinuous, non-convex and would require computationally intensive techniques. To tackle this issue, we propose a solution using a different approach based on a procedure that resembles *importance sampling*. This new approach provides a general and flexible framework for gene selection as well as for other model selection problems.

The advantages of this proposal are multiple:

- **Flexibility**: It allows the users to specify a criterion that can be tailored to the specific problem setting. It is able to handle different kinds of responses, problems of missing and contaminated data, multicollinearity, etc.
- **Prediction Power**: The result of the procedure is a set of models with high predictive power with respect to the specified criterion. It is especially suitable in selecting genes and models to achieve accurate predictions.
- **Dimension-reduction**: It can provide an assessment of the dimension of the problem because it greatly reduces the number of necessary covariates and eases the interpretation without requiring any preliminary size reduction.
- **Network-building**: With the reduced model size, it preserves the capacity to build gene-networks to provide a more general view of the potential paradigmatic structures of the genetic information.

This last aspect is of great interest for gene selection since this list can provide insight into the complex mechanisms behind different biological phenomena. Different cases, some of which can be found in Section 4, indicate that this method appears to outperform other methods in terms of criteria minimization while, at the same time, selects models of considerably smaller dimension which allow improved interpretation of the results. The set of selected models can naturally be viewed as a network of possible structures of genetic information. We call this a paradigmatic network. In Section 4 we give an example of a graphical representation of such networks based on the analysis of one of two cancer data sets which are discussed therein.

In this paper we first describe and formalize the proposed approach within the model selection statistical framework in Section 2. In Section 3 we illustrate the techniques and algorithms used to address the criterion minimization problem highlighted in Section 2. The performance of our approach is then illustrated on two data sets concerning leukemia classification (Golub et al., 1999) and breast cancer classification (Chin et al., 2006), and in simulation study, in Section 4 and Section 5 respectively. We conclude the paper in Section 6 by summarizing the benefits of the new approach and providing an outlook on other potential applications that can benefit from this methodology.

## 2. Approach

To introduce the proposed method, let us first define some notation which will be used throughout this paper:

1. Let J_*f*_ = {1, 2, …, *p*} be the set of indices for *p* potential covariates included in the *n* × *p* matrix **X**. We allow **X** to include a vector of 1s.
2. Let J = P(J_*f*_)\∅, |J| = 2^*p*^ − 1, be the power set including all possible models that can be constructed with the *p* covariates excluding the empty set.
3. Let *J* ∈ J be a model belonging to the above mentioned power set.
4. Let **β**^*J*^ ∈ ℝ^*p*^ be the parameter vector for model *J*, i.e.,

βkJ={βkif  k∈J0if  k∈J

where $\beta_{k}^{J}$, **β**_*k*_ are respectively the *k*th element of **β**^*J*^ and **β**, with $\beta ={\left(\beta_{1},\ldots ,\beta_{p}\right)}^{T}\in B\subseteq {\mathbb{R}}^{p}$.

Keeping this notation in mind, for a given model *J* ∈ J we have that

(1) $$𝔼[Y|X]=g(X,\beta^{J}),$$

where 𝔼[·] is the expectation operator and *g*(·, ·) is a link function known up to the parameter vector **β**^*J*^ ∈ ℝ^*p*^. Models of the form (1) are very general and include all parametric models and a large class of semiparametric models when *g*(·, ·) is not completely known.

We assume that for a fixed *J*, based on a specific choice for model (1) with corresponding parameter vector **β**^*J*^ and given a new covariate vector **X**_0_, the user can construct a prediction $\hat{Y}\left({\text{X}}_{0},\beta^{J}\right)$. To assess the quality of this prediction we assume that we have a divergence measure available which we denote as $D\left\{\hat{Y}\left({\text{X}}_{0},\beta^{J}\right),Y_{0}\right\}$. The only requirement imposed on the divergence measure is that it satisfies the property of positiveness, i.e.,

$$\begin{array}{l} D(u,v)>0\text{ for }u\neq v\\ D(u,v)=0\text{ for }u=v. \end{array}$$

With this property being respected, the divergence measure can arbitrarily be specified by the user according to the interest in the problem. Examples of such divergence measures include the *L*_1_ loss function

$$D\{\hat{Y}(X_{0},\beta^{J}),\;Y_{0}\}=\;|\hat{Y}(X_{0},\beta^{J})-Y_{0}|$$

or an asymmetric classification error

$$\begin{array}{l} D\{\hat{Y}(X_{0},\beta^{J}),\;Y_{0}\}=\;I\{\hat{Y}(X_{0},\beta^{J})=1,Y_{0}=0\}w_{1}\\ \;+\;I\{\hat{Y}(X_{0},\beta^{J})=0,Y_{0}=1\}w_{2}. \end{array}$$

where *w*_1_, *w*_2_ ≥ 0. The latter is for a Bernoulli response and is typically an interesting divergence measure when asymmetric classification errors have to be considered. Indeed, in most clinical situations, the consequences of classification errors are not equivalent with respect to the direction of the misclassification. For instance, the prognosis and the treatment of Estrogen Receptor (ER) positive Breast Cancers (BC) are quite different from those of ER negative ones. Indeed, if a patient with ER negative is treated with therapies designed for patients with ER positive, the consequence is much more severe than if this were done the other way round because of the excessive toxicities and potentially severe side effects. It therefore makes sense to give different values to *w*_1_ and *w*_2_. By defining *w*_1_ > *w*_2_ we would take these risks into account, where *w*_1_ would be the weight for a misclassification from ER negative to ER positive BC and *w*_2_ for the opposite direction. Weight values can be modulated according to the current medical knowledge and the clinical intuition of the physicians.

Considering this divergence measure *D*(·, ·), we are consequently interested in finding the best models within the general class given in Equation (1). To do so, we would ideally aim at solving the following risk minimization problem :

(2) $${\hat{\beta }}^{J}\in B\equiv \underset{J\in J}{\mathrm{argmin}}\;\underset{\beta^{J}}{\mathrm{argmin}}\;{𝔼}_{0}\left[D\left\{\hat{Y}(X_{0},\beta^{J}),Y_{0}\right\}\right],$$

where 𝔼_0_ denotes the expectation on the new observation (*Y*_0_, **X**_0_). Let *J*_0_ denote the models with the smallest cardinality among all ${\hat{\beta }}^{J}\in B$. Note that there could be more than one model with the same prediction property and of the same size, hence *J*_0_ could contain more than one model. Let us define the models corresponding to *J*_0_ as the “true” models. Thus, our “true” models are essentially the most parsimonious models that minimize the expected prediction error.

The optimization problem in Equation (2) is typically very difficult to solve. First of all, supposing we do not consider interaction terms, the outer minimization would require to compare a total of 2^*p*^ − 1 results, each a result of the inner minimization problem. In addition, each of the 2^*p*^ − 1 inner minimization problems is also very hard to solve, even if the risk ${𝔼}_{0}\left[D\left\{\hat{Y}\left({\text{X}}_{0},\beta^{J}\right),Y_{0}\right\}\right]$ were a known function of **β**^*J*^. Indeed, the inner minimization problem is in general non-convex and could be combinatorial, implying that the minimizer might not be unique. For example, when *D*(·, ·) is the classification error, this problem is combinatorial by nature. In practice, the computational challenge is even greater because the risk function ${𝔼}_{0}\left[D\left\{\hat{Y}\left({\text{X}}_{0},\beta^{J}\right),Y_{0}\right\}\right]$ is a function of **β**^*J*^ without explicit form and needs to be approximated.

We propose to estimate ${𝔼}_{0}\left[D\left\{\hat{Y}\left({\text{X}}_{0},\beta^{J}\right),Y_{0}\right\}\right]$ via an *m*-fold cross-validation (typically *m* = 10) repeated *K* times. More specifically, for a sample of size *n*, at the *k*th repetition we randomly split the data into *m* subsets *I*_*k, l*_ of size *n*_*l*_ for *l* = 1, …, *m*. Given this, the estimated risk is

(3) $${\hat{𝔼}}_{0}\left[D\left\{\hat{Y}(X_{0},\beta^{J}),Y_{0}\right\}\right]=\frac{1}{mK}\sum_{k\;=\;1}^{K}\sum_{l\;=\;1}^{m}\frac{1}{n_{l}}\sum_{i\in I_{k,l}}D\{\hat{Y}(X_{i},\beta^{J}),Y_{i}\}.$$

Having approximated the expectation 𝔼_0_, the minimization problem in Equation (2) becomes

(4) $$\underset{J\in J}{\mathrm{argmin}}\;\underset{\beta^{J}}{\mathrm{argmin}}\;{\hat{𝔼}}_{0}\left[D\left\{\hat{Y}(X_{0},\beta^{J}),Y_{0}\right\}\right].$$

Despite the above approximation, the minimization problem remains complicated for the reasons mentioned earlier. Thus, we further eliminate the inner minimization problem in Equation (4) by inserting an estimator ${\hat{\beta }}^{J}$ obtained independently from the minimization procedure. More specifically, we assume that an estimator of **β**^*J*^, say ${\hat{\beta }}^{J,k}$, is available based on model (1) and “training” observations containing all the observations except those in *I*_*k, l*_. This estimator can be any available estimator, for example, the maximum likelihood estimator (MLE), a moment based estimator, or a quantile regression based estimator, etc. (see for example, Azzalini, 1996; Hall, 2005; Koenker, 2005). We then replace the inner minimization in Equation (4) directly with the approximate expectation evaluated at ${\hat{\beta }}^{J,k}$'s and simplify Equation (4) to

(5) $$\underset{J\in J}{\text{argmin}}\;\frac{1}{mK}\sum_{k=1}^{K}\sum_{l=1}^{m}\frac{1}{n_{l}}\sum_{i\in I_{k,l}}D\{\hat{Y}(X_{i},{\hat{\beta }}^{J,k}),\;Y_{i}\}.$$

The intuition of replacing the inner minimization in Equation (4) with a sample average evaluated at an arbitrary estimator is due to the fact that this estimator, under a fixed “true” model and regardless of whether this estimator is a standard MLE or a minimizer of the divergence measure *D*(·, ·), is an approximation to the “true” parameter. This means that, consequently, different estimators are “close” to each other. As a consequence, the minimization problem in Equation (5) can be considered to be a close approximation to $\underset{\beta^{J}}{\min}E_{0}\left[D\left\{\hat{Y}\left({\text{X}}_{0},\beta^{J}\right),Y_{0}\right\}\right]$. In fact, using an informal law of large numbers argument, as *n*_*l*_ → ∞, then we have that ${\hat{\beta }}^{J}\overset{p}{\to }\beta^{J}$. If in addition *m* → ∞ then, under some regularity conditions on *D*(·, ·), the averages tend to the desired expectation. On the other hand, if instead we consider *m* as fixed, we would have an unbiased estimator of the expected risk. A thorough development of these arguments goes beyond the scope of this paper.

We now have an optimization problem in Equation (5) which requires a comparison of 2^*p*^ − 1 values and is much easier to solve. To further reduce the number of comparisons, the following section describes some procedures and algorithms allowing to solve this problem in a more efficient manner.

## 3. Heuristic procedure

To solve the optimization problem in Equation (5), we propose an approach designed to have the following three features:

1. Identify a **set of models** that carry large predictive power instead of a single “best” model;
2. Find this set of models within a **reasonable time**, without having to explore all possible models;
3. This set achieves **sparsity**, i.e., most of the parameters in **β** will be fixed at zero in each of the models in the set.

Note that the last feature above reflects the belief that most of the covariates are irrelevant for the problem under consideration and should be excluded. Indeed, our method is designed to work effectively if such a sparsity assumption holds, putting it on the same level of almost all variable selection procedures in the literature. Moreover, we require the method to have the first feature in order to increase flexibility in terms of interpretation. Indeed, in many domains such as gene selection, for example, the aim may not be to find a single model but a set of variables (genes) that can be inserted in a paradigmatic structure to better understand the contribution of each of them via their interactions.

Given this goal, assume that we have at our disposal an estimate of the measure of interest *D*(·, ·) for all possible 2^*p*^ − 1 models. In this case, our interest would be to select a set of “best” models by simply keeping the set of models that have a low discrepancy measure *D*(·, ·). It is of course unrealistic to obtain a discrepancy measure for all models in most practical cases because this would require a considerable amount of time for computation. Therefore, in order to achieve the second feature, instead of examining all possible models, we can randomly sample covariates from J. The random sampling needs to be carefully devised because in practice, for example in gene selection problems, the number of covariates *p* can easily reach thousands or tens of thousands (see examples in Section 4, where *p* = 7129 and *p* = 22, 215 respectively). In such situations, 2^*p*^ − 1 is an extremely large number and the probability of randomly sampling a “good” set of variables from the 2^*p*^ − 1 variables is very small. Using the sparsity property of the problem, we propose to start with the set of variables M_0_ (typically an empty set) and increase the model complexity stepwise. Throughout this procedure, we ensure that at step *k*, the most promising covariates based on the evaluation at step *k* − 1 are given higher probabilities of being randomly drawn. The last idea is in the spirit of “importance sampling” in the sense that covariates with more importance based on the previous step are “encouraged” to be selected in the current step. Note that by construction we achieve sparsity if we stop the stepwise search at models of size *d*_max_ ≪ *p*.

More formally, let us first define the set of all possible models of size *d* as

$$S_{d}=\{(i_{1},\ldots ,i_{d})\;|\;i_{1},\ldots ,i_{d}\in J_{f};\;i_{1}<\ldots <i_{d}\}.$$

We then define the set of promising models, $S_{d}^{*}$, as the ones with an estimated out-of-sample divergence measure *D*(·, ·) below a certain estimated α-quantile. The value of α is user-defined depending on the problem at hand, and is typically a small value such as α = 1%. The formal definition of this set would then be

$$S_{d}^{*}=\{J\;|\;J\in S_{d}\;;\;{\hat{D}}_{J}\leq {\hat{q}}_{d}(\alpha )\},$$

where

(6) $${\hat{D}}_{J}\equiv \frac{1}{mK}\sum_{k=1}^{K}\sum_{l=1}^{m}\frac{1}{n_{l}}\sum_{i\in I_{k,l}}D\{\hat{Y}(X_{i},{\hat{\beta }}^{J,k}),\;Y_{i}\},$$

and ${\hat{q}}_{d}\left(\alpha \right)$ is the α-quantile of the ${\hat{D}}_{J}\left(J\in S_{d}\right)$ values issued from *B* randomly selected models. Finally, we define the set of indices of covariates that are in $S_{d}^{*}$ as

$$I_{d}^{*}=\{i\;|\;i\in J,\;J\in S_{d}^{*}\}$$

whose complement we define as $I_{d}^{c}$ (i.e., all those covariates that are not included in $I_{d}^{*}$).

With this approach in mind and using the above notations, to start the procedure we assume that we have *p* variables from which to select.

- *Initial Step:* We start by adding the number of variables *d* = 1 to our initial variable set M_0_ with the goal of finally obtaining the set $I_{1}^{*}$.
  1. Construct the *p* possible one variable models by augmenting M_0_ with each of the *p* available variables.
  2. Compute $\hat{D}$ for every model obtained in Step A.1.
  3. From Steps A.1 and A.2, construct the set $I_{1}^{*}$ using Equation (3). Go to Step B and let *d* = 2.
- *General Step:* We define here the general procedure to construct $I_{d}^{*}$ for 2 ≤ *d* ≤ *d*_max_.
  1. Augment M_0_ with *d* variables as follows:
    - Randomly select a set, either set $I_{d-1}^{*}$ with probability π or its complement $I_{d-1}^{c}$ with probability 1−π.
    - Select one variable uniformly at random and without replacement from the set chosen in Step (i) and add this variable to M_0_.
    - Repeat Steps (i) and (ii) until *d* variables are added to M_0_.
  2. Construct a model of dimension *d* using the *d* variables selected in Step B.1. Repeat Step B.1 *B* times to construct *B* such models.
  3. From Steps B.1 and B.2, construct the set $I_{d}^{*}$ according to Equation (3). If *d* < *d*_max_, go to Step B and let *d* = *d* + 1, otherwise exit algorithm.

Once the algorithm is implemented, the user obtains an out-of-sample discrepancy measure for all evaluated models. Given that the goal is to obtain a set of models $S_{d}^{*}$ with high predictive power, the discrepancy measure delivers the criterion based on which it is possible to determine the optimal model dimension and the corresponding network structure.

### 3.1. Practical considerations

The algorithm described above lays out the basic procedure to solve the problem in Equation (2). However, as many other heuristic selection procedures, there are a series of “hyper-parameters” to be determined and certain aspects to be considered. In the following paragraphs we will discuss some of these issues arising when implementing our algorithm in practice.

#### 3.1.1. Choice of algorithm inputs

The parameters *d*_max_, *B*, α and π of the above algorithm are to be fixed by the user. As mentioned earlier *d*_max_ represents a reasonable upper bound for the model dimension which is constrained to *d*_max_ ≤ *L*, where *L* depends on the limitations of the estimation method and is commonly the sample size *n*. As for the parameter *B*, a larger value is always preferable to better explore the covariate space. However, a larger *B* implies heavier computations, hence a rule of thumb that could be used is to choose this parameter such that $p\;\leq \;B\;\leq \;\left(\frac{p}{2}\right)$. As mentioned earlier, the parameter α should define a small quantile, typically 1%. Finally, π determines to what extent the user assigns importance to the variables selected at the previous step. Given that *d*_max_ ≪ *p* and α is small, we will typically have that $\left|I_{d-1}^{*}|<|I_{d-1}^{c}\right|$. In this setting, a choice of π = 0.5 for example would deliver a higher probability for the variables in $I_{d-1}^{*}$ to be included in $I_{d}^{*}$. All other parameters being equal, increasing the value of π would decrease the probability of choosing a variable in $I_{d-1}^{c}$ and vice versa. Moreover, we discuss in Appendix A (Supplementary Material) how the proposed algorithm can be adjusted to situations where *p* is either small or very large.

As a final note, it is also possible for the initial model M_0_ to already contain a set of *p*_0_ covariates which the user considers to be essential for the final output. In this case, the procedure described above would remain exactly the same since the procedure would simply select from the *p* covariates which are not in the user-defined set and the final model dimension would simply be *p*_0_ + *d*.

#### 3.1.2. Model dimension and network building

The final goal of the algorithm is to find a subset of models of dimension d^*^ that in some way minimize the considered discrepancy. A possible solution would be to select the set of models $S_{d^{*}}^{*}$ such that $d^{*}=\min_{\in \left\{1,\ldots ,d_{\text{max}}\right\}}q_{d}\left(\alpha \right)$. However, the quantity *q*_*d*_(α) is unknown and replaced by its estimator ${\hat{q}}_{d}\left(\alpha \right)$. Due to this, a solution that might be more appropriate would be to consider a testing procedure to obtain d^*^ taking into account the variability of ${\hat{q}}_{d}\left(\alpha \right)$. For example, we could find the dimension d^*^ such that we cannot reject the hypothesis that ${\hat{q}}_{d^{*}}\left(\alpha \right)={\hat{q}}_{d^{*}+1}\left(\alpha \right)$. Thus we sequentially test whether ${\hat{q}}_{j+1}$ is smaller than ${\hat{q}}_{j}$ for *j* = 1, …, *d*_max_. As long as the difference is significant we increment *j* by one unit, otherwise the minimum is reached and d^*^ = *j*.

The type of test and its corresponding rejection level are determined by the user based on the nature of the divergence measure. For example, if we take the *L*_1_ loss function as a divergence, one could opt for the Mann-Whitney test or if the loss function is a classification error (as in the applications in Section 4), one could choose the binomial test or other tests for proportions. The rejection level will depend, among others, on the number of tests that need to be run, typically less than *d*_max_ − 1, and need to be adjusted using, for example, the Bonferroni correction. Finally, once the set $S_{d^{*}}^{*}$ is obtained, the user may still want to “filter” the resulting models. Indeed, the number of models in the solution $S_{d^{*}}^{*}$ may be large and the corresponding divergence estimates may vary considerably from model to model. Since these divergence measures are estimators, we again propose a multiple testing procedure to reduce the number of models in $S_{d^{*}}^{*}$. Before doing so, we eliminate redundant models, thereby making sure that every model is included only once. Then, we start the testing procedure with an empty set $S_{d^{*}}^{0}=\emptyset$ to which we add the model (or one of the models) that has the minimum divergence measure estimate, denoted ${\hat{D}}_{J_{\text{min}}}$, where $J_{\text{min}}\in S_{d^{*}}^{*}$ denotes this model. Then for every model $J\in S_{d^{*}}^{*}\backslash J_{\text{min}}$, we test whether ${\hat{D}}_{J}$ is greater than ${\hat{D}}_{J_{\text{min}}}$. We add the model to $S_{d^{*}}^{0}$ if the difference is not significant and stop adding models as soon as the test deems that the divergence of the next model is indeed larger. By doing so we finally obtain $S_{d^{*}}^{0}\subseteq S_{d^{*}}^{*}$ which is the set containing the models (and hence covariates) which can be interpreted in a paradigmatic network. Generally speaking, this network can be built starting from the most frequent covariate(s) present in $S_{d^{*}}^{0}$ (we call these “hubs”) and, subsequently, connecting these with the most frequent covariates included in the models with the previous hubs. This can be continued until the number of connected hubs is equal to d^*^.

### 3.2. Related literature

Some of the ideas put forth in this work have also been considered in the literature. An extensive survey of the related works goes beyond the scope of this paper. Here we briefly describe some of the connections to three main ideas that have been explored to this point.

The first one is recognizing that practitioners might aim to minimize some criterion that differs from likelihood-type losses. An interesting paper illustrating this point is Juang et al. (1997) in the context of speech recognition. For their classification problem, these authors propose to minimize a “smoothed” version of the decision rule used for classification. The advantage of this procedure is that it yields better misclassification errors than using pure likelihood based criteria which intrinsically fit a distribution to the data. In the approach presented in this work we also deliver an approximate solution but, as opposed to approximating the problem and solving the latter in an exact manner as in Juang et al. (1997), we define the exact problem and try to approximately minimize the misclassification error through our algorithm.

Secondly, there is a large literature that uses stochastic search procedures to explore the space of candidate models. Influential work in this direction includes George and McCulloch (1993) and George and McCulloch (1997) who postulate hierarchical Bayesian models. In their set-up, subsets of promising predictors form models with higher posterior probabilities. An interesting application of this framework for disease classification using gene expression data is the work of Yang and Song (2010). Cantoni et al. (2007) also consider a random exploration of the space of possible models, but avoiding the Bayesian formulation of George and McCulloch (1993). Their approach defines a probability distribution for the various candidate models based on a cross-validated prediction error criterion and then uses a Markov Chain Monte-Carlo method to generate a sample from this probability distribution. An important feature of the stochastic search implied by our algorithm is that it is a greedy method, while the aforementioned methods are not. The typical forward/backward greedy algorithms proposed in the literature are not random, while existing stochastic procedures are not greedy. Thus, the combination of greedy approach and random search approach seems to be new (see for instance Zhang, 2011, for some theory on greedy algorithms in sparse scenarios).

Finally, other authors have also considered providing a set of interesting models as opposed to a single “best” model. The stochastic search procedures mentioned in the above paragraph can naturally be used to obtain a group of interesting models. For example, Cantoni et al. (2007) consider a set of best indistinguishable models in terms of prediction. Random forests can be used to select variables and account for the stability of the chosen model as in Díaz-Uriarte and De Andres (2006). These methods can also be used to construct a set of interesting models. It is also worth noting that typical ensemble methods, such as bagging and boosting (Friedman et al., 2010), exploit multiple models mainly to yield good predictions. In our approach however not only can the different models that we explore be averaged for prediction, but typically each of them will give good predictions, be sparse and easy to interpret, and together can be used to construct a network.

## 4. Case studies

In this section we provide an example of how the methodology proposed in this paper selects and groups genes to explain, describe and predict specific outcomes. We focus on the data-set (hereinafter *leukemia*) which collects information on Acute Myeloid Leukemia (AML) and Acute Lymphoblastic Leukemia (ALL) and is frequently used as an example for gene selection procedures. Indeed, Golub et al. (1999) were among the first to use this data to propose a gene selection procedure which was then followed up by other proposals that used the same data to compare their performance. We will use this data-set to underline the features and advantages of the proposed method. A second data-set concerning the research on breast cancer (presented in Chin et al., 2006) is analyzed in Appendix C (Supplementary Material) to show the outputs of the proposed method from another example. Note that both he *Leukemia* (Section 4.1) and the *Breast Cancer* (Appendix C in Supplementary Material) data-sets are made available in the R package “datamicroarray”.

The analysis of these data-sets focuses both on the advantages of the proposed methodology and the biological interpretation of the outcomes. One of the goals of our method is to help decipher the complexity of biological systems. We will take on an overly simplified view of the cellular processes in which we will assume that one biomarker maps to only one gene that in turn has only one function. Although this assumption is not realistic, it allows us to give a straightforward interpretation of the selected models or “networks” which can therefore provide an approximate first insight into the relationships between variables and biomarkers (as well as between the biomarkers themselves). We clarify that we do not claim any causal nature in the conclusions we present in these analyses but we believe that the selected covariates can eventually be strongly linked to other covariates that may have a more obvious and direct interpretation for the problem at hand. Finally, the data-set has binary outcomes [as does the data-set in Appendix C (Supplementary Material)], hence we will make use of the Classification Error (CE) as a measure of prediction performance and we will not assign weights to a given prediction error. This means that misclassification errors are given the same weight, in the sense that a false positive prediction (e.g., predicted “presence” when the truth is “absence”) is considered as undesirable as a false negative prediction. However, our method can also consider divergence measures based on unequal weights as highlighted in Section 2.

### 4.1. Acute leukemia

Golub et al. (1999) were among the first to propose an automatic selection method for cancer classification and demonstrated the advantages of using such a method. One of the main applications of their method was on the *leukemia* data-set in which information regarding 72 patients is included, namely their type of leukemia (25 patients with AML and 47 patients with ALL) and 7129 gene expressions used as explanatory variables to distinguish between two types of leukemia. As explained in Golub et al. (1999) this distinction is critical for successful treatment which substantially differs between classes. In fact, although remissions can be achieved using any of these therapies, cure rates are markedly increased and unwarranted toxicities are avoided when targeting the specific type of leukemia with the right therapy.

#### 4.1.1. Statistical analysis

**Figure 1:**
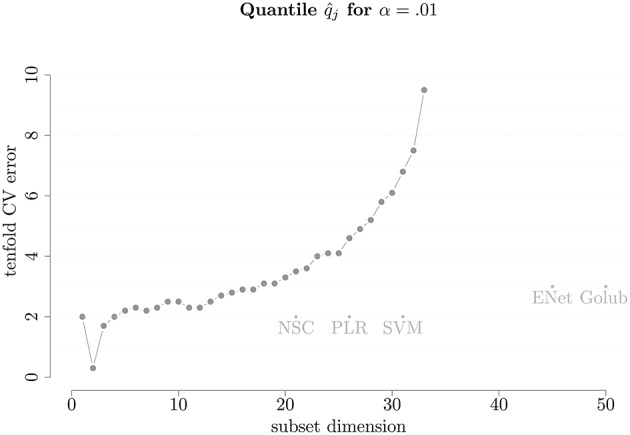
**Number of covariates vs. $\hat{D}$ on leukemia cancer classification training set**. The names are abbreviations for other selection method referred to in Table 1.

In order to understand how our proposed methodology performs compared to existing ones, we split the *leukemia* data into the same training set (38 patients) and test set (34 patients) as in the original work by Golub et al. (1999). We employ our method on the training set to understand the dimension of the model and to select the most relevant genes. Setting α = 0.01, the corresponding observed quantile of the 10-fold cross-validation CE ($\hat{D}$) is shown in Figure 1. It can be seen that the error immediately decreases to almost zero when using two covariates instead of one, after which it roughly monotonically increases, suggesting that the optimal model dimension is two.

In Figure 1 we also plotted the performance of the other selection methods used on this training data which are represented by labeled dots reporting the acronyms of these methods that are listed in Table 1. These cross-validation errors are taken from Zou and Hastie (2005) in the same setting in which we ran the proposed method. However, another table in which the competing methods were ran using currently available software is presented in Appendix B (Supplementary Material) where the conclusions in terms of comparison do not differ from those presented in Table 1,^1^. Indeed, the approach proposed in this work compares favorably to all other methods in terms of prediction power since they lie under the curve to the right of its minimum indicating that, compared to our method, they select models of considerably higher dimensions without achieving the same degree of performance in terms of CE. Therefore, for this particular case, our method outperforms the other methods. The sparsity and tenfold CV error are further illustrated in Table 1, where we also present the average prediction error on the test data. Considering the latter, it can be seen how the performance of the different methods are similar but the proposed method (which we refer to as *Panning*) is able to achieve the same performance by selecting models of a considerably lower dimension. As a final note to the table, the last line reports the performance of model averaging. Indeed, if the interest lies in predicting, the algorithm of Section 3 provides a set of models whose CE is below a given quantile α. The predictions of these models can be used in the spirit of model averaging where a general prediction can be obtained by taking the average of predictions of the selected set of models. The proposed methodology can therefore be potentially seen as a bridge between model selection and model averaging.

**Table 1:** **Summary of Leukemia classification results**.

**Method**	**Tenfold CV error**	**Test error**	**Number of genes**
Golub	3/38	4/34	50
Support vector machine	2/38	1/34	31
(with recursive feature elimination)			
Penalized logistic regression	2/38	1/34	26
(with recursive feature elimination)			
Nearest shrunken centroids	2/38	2/34	21
Elastic net	3/38	0/34	45
Panning Algorithm (107)			
Model a	0/38	2/34	2
Model b	0/38	2/34	2
Model c	0/38	2/34	2
[…]			
Model averaging		2/34	2

*The table is taken from where we added the Panning Algorithm.We obtained a total of 107 models of size 2 (109 different biomarkers) using a probability α=*0.01*, *B*=*20'000* bootstrap replicates, a selection probability π=*0.5* with *D*(·,·) estimated through tenfold-CV repeated *K*=*10* times. Models “a” to “c” are three examples out of the 107 models. All 107 models have a tenfold-CV error of 0. The best test error is 2 and the worst is 12. For model averaging all models are equally weighted*.

**Figure 2:**
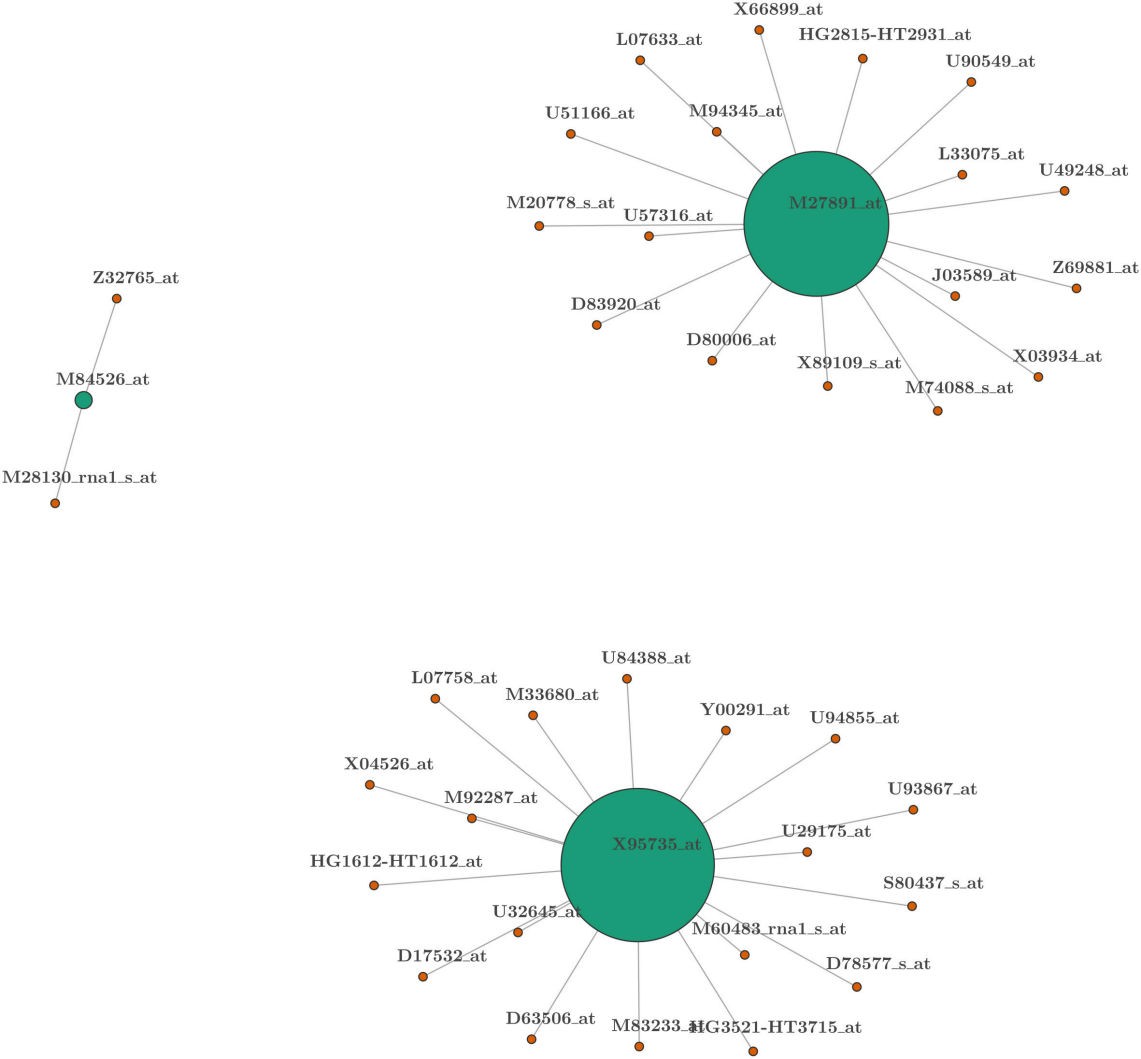
**Network representation of biomarkers selected from the *leukemia* data-set**. Colors represent the position of covariates within the model: green for first position (hub) and orange for second. The width of the connecting lines is proportional to the frequency with which two biomarkers appear in the same model. The size of the disk is proportional to the frequency with which a biomarker is present within the selected set of models.

Once this procedure is completed, we can create a gene network to facilitate interpretation. This is a direct benefit of our method which does not deliver a single model after the selection process but provides a series of models that can be linked to each other and interpreted jointly. Indeed, the existence of a single model that links the covariates to the explained variable is probably not realistic in many settings, especially for gene classification. For this reason, the frequency with which each gene is included within the selected models and with which these genes are coupled with other genes provides the building block to create an easy-to-interpret gene network with powerful explanatory and predictive capacities. A graphical representation of this gene network can be found in Figure 2 where the size of a disk represents the frequency with which a particular biomarker is included in the selected models, and the line connecting the disks indicates the biomarkers that are included in the same model. Since the model dimension in this case is two, each biomarker is connected with only one other biomarker and, as can be observed, the proposed method identifies three main “hubs” for the networks (green disks) generating three networks. Appendix B (Supplementary Material) also reports a related table where the biomarkers are listed according to their position in the model. These positions represent families of biomarkers (or genes) whose members are interchangeable. By the latter we mean that, given the presence of biomarkers from other families, specific biomarkers can be replaced by another biomarker from within the same family without losing predictive power. This is the idea behind finding a paradigmatic network for gene selection purposes. In the following paragraph we provide a summary biological interpretation of the the three main biomarkers (i.e., the most frequent in the selected models) which we call “hubs” from which the networks start.

#### 4.1.2. Biological interpretation

The three hubs that were identified are the following:

1. Cystatin C: a secreted cysteine protease inhibitor abundantly expressed in body fluids (see Xu et al., 2015);
2. Zyxin: a zinc-binding phosphoprotein that concentrates at focal adhesions and along the actin cytoskeleton;
3. Complement factor D: a rate-limiting enzyme in the alternative pathway of complement activation (see White et al., 1992).

In the current state of knowledge about acute leukemia, these three hubs appear to make sense from a biological viewpoint. Cystatin C is directly linked to many pathologic processes through various mechanisms and recent studies indicate that the roles of Cystatin C in neuronal cell apoptosis induction include decreasing B-cell leukemia-2 (BCL-2) whose deregulation is known to be implicated in resistant AML (see Sakamoto et al., 2015). Zyxin is a protein that interacts with Vasodilator-stimulated phosphoprotein (VASP) with both being involved in cellular adhesion and motility. VASP interacts with ABL (breakpoint cluster region-abelson) and is a substrate of the BcrAbl oncoprotein which drives oncogenesis in patients with chronic myeloid leukemia (CML) due to a constitutive activation of tyrosine kinase activity (see Bernusso et al., 2015). Further results suggest that the phosphorylation and dephosphorylation cycle of VASP by the Abi-1-bridged mechanism regulates association of VASP with focal adhesions, which may regulate adhesion of Bcr-Abl-transformed leukaemic cells (see Masahiro et al., 2012). Finally, Complement factor D, together with several other components of both the classical and alternative complement cascade, is primarily expressed through both adipocytes and monocytes-macrophages in human subjects (see White et al., 1992; Gabrielsson et al., 2003). A recent review in Ratajczak (2014) has stressed the role of the complement cascade as a trigger for hematopoietic stem cells from bone marrow into blood.

The interpretation of the network can be carried out through plots or tables such as those presented in Appendix B (Supplementary Material) where the biomarkers can be grouped together into clusters having the same biological traits, e.g., transcription/translation factor activity, DNA repair and catabolism, apoptotic activity. This grouping allows a more straightforward interpretation of the links between the different families thereby providing a more general overview of how the elements of the identified network interact.

## 5. Simulation study

In this section we present a simulation study whose goal is to highlight the practical benefits of the proposed method over competing methods frequently used in genomics. Considering the complexity of simulating from a gene network, in this setting we limit ourselves to considering the existence of a unique true model which therefore does not allow to assess one of the features of the proposed approach which is its network building capacities. Hence, this section specifically focuses on the prediction power and dimension-reduction ability of the method and, for the comparison with alternative methods to be fair, we only keep one model for each simulation replicate. This means that, once the dimension of the model has been identified, the model with the lowest estimated prediction error is kept (thereby discarding the other potential candidates).

In this optic, for the simulation study we mimicked the acute leukemia dataset seen in Section 4.1 where we set the true model to be generated by a combination of two gene expressions: Cystatin C (*X*_1_) and Thymine-DNA Glycosylase (*X*_2_) (see Section 4.1.2). Hence the response *y*^⋆^ in the simulations is a realization of a Bernoulli random variable with probability parameter γ which is obtained through a logit-link function applied to a linear combination of the two above-mentioned variables plus an intercept (with all β coefficients equaling one) i.e.,:

$$\gamma =\frac{1}{1+\exp^{\left(1+X_{1}+X_{2}\right)}}.$$

Once the binary response variable *y*^⋆^ is generated, this is then separated into a training and a test set of the same size as that in the original data-set (i.e., 38 and 34 respectively).

Using the implementation of the proposed algorithm available at the corresponding GitHub repository^2^, the results of the simulations based on 100 replications can be found in Table 2 where the median performances are reported. The proposed algorithm's hyper-parameters are α = 0.01, *B* = 20′000, π = 0.5 and *D*(·, ·) based on the classical tenfold-CV (*K* = 1). To select the dimension d^*^, we ran the testing procedure described in Section 3.1.2 based on a *p*-value of 0.1. As mentioned earlier, unlike Table 1, we only kept one model of dimension d^*^ instead of a set of models. This model was chosen such that it had the minimum training error and, if this minimum was not unique, then the model was randomly chosen among those achieving this minimum.

**Table 2:** **Median performances of selection methods on 100 simulations based on a dataset of 7129 genes where only two are relevant**.

**Method**	**Tenfold CV error**	**Test error**	**Number of genes**
Panning algorithm	$\underset{\left(\text{all}\right)}{0∕38}$	$\underset{\left(\text{min}:0∕34;\text{max}:12∕34\right)}{1∕34}$	$\underset{\left(\text{all}\right)}{2∕7129}$
Elastic net	$\underset{\left(\text{min}:0∕38;\text{max}:8∕38\right)}{0∕38}$	$\underset{\left(\text{all}\right)}{0∕34}$	$\underset{\left(\text{min}:1;\text{max}:104\right)}{81∕7129}$
Support vector machine	$\underset{\left(\text{all}\right)}{0∕38}$	$\underset{\left(\text{all}\right)}{15∕34}$	$\underset{\left(\text{min}:4;\text{max}:6\right)}{4∕7129}$
Penalized logistic regression	$\underset{\left(\text{min}:0∕38;\text{max}:4∕38\right)}{3∕38}$	$\underset{\left(\text{min}:8∕34;\text{max}:12∕34\right)}{12∕34}$	$\underset{\left(\text{all}\right)}{5∕7129}$
Logistic regression	$\underset{\left(\text{min}:0∕38;\text{max}:4∕38\right)}{1∕38}$	$\underset{\left(\text{min}:2∕34;\text{max}:3∕34\right)}{3∕34}$	$\underset{\left(\text{all}\right)}{2∕7129}$
Nearest shrunken centroids	$\underset{\left(\text{min}:7∕38;\text{max}:18∕38\right)}{12∕38}$	$\underset{\left(\text{min}:0∕34;\text{max}:5∕34\right)}{5∕34}$	$\underset{\left(\text{min}:3;\text{max}:30\right)}{30∕7129}$

Concerning the competing methods, these were implemented using existing R functions with default values. For the Elastic Net we used the R package “glmnet”, that implements the coordinate descent algorithm described in Friedman et al. (2010), using the cv.glmnet() function to select the lasso parameter. We performed a grid search over the values {0.2, 0.4, 0.6, 0.8, 1} for the parameter α of the Elastic Net and kept the value yielding the best deviance^3^. As for the Nearest Shrunken Centroids method of Tibshirani et al. (2002) we considered the R package “pamr”. We applied the function pamr.train() on the training data and took the value of the tuning parameter (threshold) yielding the best classification. The Support Vector Machines approach with recursive feature elimination was obtained through the function fit.rfe() in the “pathClass” R package. We used the function crossval() to select the soft-margin tuning parameter discussed in Chapelle et al. (2002). Finally, the penalized *L*2 logistic regression with greedy forward selection and backward deletion was implemented with the function step.plr() of the “stepPlr” R package. Note that this function also considers all possible interactions among the active variables and it is an implementation of the methodology proposed by Park and Hastie (2008). Finally, we used our own implementation for the logistic regression with greedy forward selection, choosing the model with the minimum BIC.

Table 2 shows how the proposed method compares favorably in terms of median performance with the respect to the competing methods. Indeed, it is the best approach (or it is among the best) both in terms of cross-validation error as in terms test error. Even considering its maximum test error it is comparable to the other methods, keeping in mind that it selects models of extremely low (and above all correct) dimensions. For example, the Elastic Net is the without doubt the best in terms of test error but it selects a unique model of size 81 (in median) making its genetic interpretation much more complex. On the other hand, the proposed algorithm selects the correct dimension and, if considering the set of best models, would deliver a network which is more straightforward to interpret.

## 6. Conclusions

This paper has proposed a new model selection method with various advantages compared to existing approaches. Firstly, it allows the user to specify the criterion according to which they would like to assess the predictive quality of a model. In this setting, it gives an estimate of the dimension of the problem, allowing the user to understand how many gene expressions are needed in a model to well describe and predict the response of interest. Building on this, it provides a paradigmatic structure of the selected models where the selected covariates are considered as elements in an interconnected biological network. The approach can handle more variables than observations without going through dimension-reduction techniques such as pre-screening or penalization.

The problem definition of this method and the algorithmic structure used to solve it deliver further advantages such as the ability to cope with noisy inputs, missing data, multicollinearity and the capacity to deal with outliers within the response and the explanatory variables (robustness).

Some issues which must be taken into account concerning the proposed method are (i) its computational demand and (ii) its need for an external validation. As far as the first aspect goes, this can be considered indeed negligible compared to the time often required to collect the data it should analyse and can be greatly reduced according to the needs and requirements of the user. Concerning the second aspect, external validation is a crucial point which is often overlooked and is required for any model selection procedure. In this sense, the proposed method does not differ from any other existing approach in terms of additional requirements.

Having proposed a method with considerable advantages for gene selection using statistical ideas in model selection and machine learning, future research aims at studying the statistical properties of this approach to understand its asymptotic behavior and develop the related inference tools.

## Author contributions

SG: Proposed the algorithm for model selection purposes and supervised the writing of the paper. NM: Adapted the algorithm for gene selection and gave biological interpretation of the algorithm outputs. RM: Programmed the basic functions for the algorithm and wrote different sections of the paper. SO: Developed the software for the algorithm to be distributed and ran the case studies with relative outputs (tables and plots). MA: Supervised the mathematical contents of the paper and provided the context for the methodology and its links with current literature. YM: Provided inputs for the formal presentation of the algorithm and guaranteed an overall quality-check.

### Conflict of interest statement

The authors declare that the research was conducted in the absence of any commercial or financial relationships that could be construed as a potential conflict of interest.

## Footnotes

^1^The use of the software making available the competing methods is described in Section 5.

^2^https://github.com/SMAC-Group/panning

^3^Note that the special cases α = 0 and α = 1 correspond respectively to ridge regression and lasso.

## References

[B1] Azzalini A. (1996). Statistical Inference Based on the Likelihood, Vol. 68 Boca Raton, FL: Chapman & Hall/CRC.

[B2] Bernusso V. A. Machado-Neto J. A. Pericole F. V. Vieira K. P. Duarte A. S. Traina F. (2015). Imatinib restores VASP activity and its interaction with zyxin in BCR–ABL leukemic cells. Biochim. Biophys. Acta 1853, 388–395. 10.1016/j.bbamcr.2014.11.008 25450971

[B3] Cantoni E. Field C. Mills Flemming J. Ronchetti E. (2007). Longitudinal variable selection by cross-validation in the case of many covariates. Stat. Med. 26, 919–930. 10.1002/sim.2572 16625521

[B4] Chapelle O. Vapnik V. Bousquet O. Mukherjee S. (2002). Choosing multiple parameters for support vector machines. Mach. Learn. 46, 131–159. 10.1023/A:1012450327387

[B5] Chin K. DeVries S. Fridlyand J. Spellman P. T. Roydasgupta R. Kuo W.-L.. (2006). Genomic and transcriptional aberrations linked to breast cancer pathophysiologies. Cancer Cell 10, 529–541. 10.1016/j.ccr.2006.10.009 17157792

[B6] Díaz-Uriarte R. De Andres S. A. (2006). Gene selection and classification of microarray data using random forest. BMC Bioinformatics 7:3. 10.1186/1471-2105-7-3 16398926 PMC1363357

[B7] Dudoit S. Fridlyand J. Speed T. P. (2002). Comparison of discrimination methods for the classification of tumors using gene expression data. J. Am. Stat. Assoc. 97, 77–87. 10.1198/016214502753479248

[B8] Friedman J. Hastie T. Tibshirani R. (2010). Regularization paths for generalized linear models via coordinate descent. J. Stat. Softw. 33:1. 10.18637/jss.v033.i01 20808728 PMC2929880

[B9] Gabrielsson B. G. Johansson J. M. Lönn M. Jernås M. Olbers T. Peltonen M.. (2003). High expression of complement components in omental adipose tissue in obese men. Obes. Res. 11, 699–708. 10.1038/oby.2003.100 12805391

[B10] George E. McCulloch R. (1993). Variable selection via gibbs sampling. J. Am. Stat. Assoc. 88, 881–889. 10.1080/01621459.1993.10476353

[B11] George E. I. McCulloch R. E. (1997). Approaches for bayesian variable selection. Stat. Sinica 7, 339–373.

[B12] Golub T. R. Slonim D. K. Tamayo P. Huard C. Gaasenbeek M. Mesirov J. P.. (1999). Molecular classification of cancer: class discovery and class prediction by gene expression monitoring. Science 286, 531–537. 10.1126/science.286.5439.531 10521349

[B13] Hall A. R. (2005). Generalized Method of Moments. Oxford: Oxford University Press.

[B14] Juang B. Hou W. Lee C. (1997). Minimum classification error rate methods for speech recognition. IEEE Trans. Speech Audio Proc. 5, 257–265. 10.1109/89.568732

[B15] Koenker R. (2005). Quantile Regression. New York, NY: Cambridge University Press 10.1017/cbo9780511754098

[B16] Kristensen V. N. Vaske C. J. Ursini-Siegel J. Van Loo P. Nordgard S. H. Sachidanandam R.. (2012). Integrated molecular profiles of invasive breast tumors and ductal carcinoma *in situ* (dcis) reveal differential vascular and interleukin signaling. Proc. Natl. Acad. Sci. U.S.A. 109, 2802–2807. 10.1073/pnas.1108781108 21908711 PMC3286992

[B17] Leng C. Lin Y. Wahba G. (2006). A note on the lasso and related procedures in model selection. Stat. Sin. 16, 1273–1284.

[B18] Masahiro M. Mizuho S. Yunfeng Y. Masayoshi I. Ryosuke F. Takuya O.. (2012). Abi-1-bridged tyrosine phosphorylation of vasp by abelson kinase impairs association of vasp to focal adhesions and regulates leukaemic cell adhesion. Biochem. J. 441, 889–899. 10.1042/BJ20110951 22014333

[B19] Park M. Hastie T. (2008). Penalized logistic regression for detecting gene interactions. Biostatistics 9, 30–50. 10.1093/biostatistics/kxm010 17429103

[B20] Ratajczak M. (2014). A novel view of the adult bone marrow stem cell hierarchy and stem cell trafficking. Leukemia 29, 776–782. 10.1038/leu.2014.346 25486871 PMC4396402

[B21] Sakamoto K. M. Grant S. Saleiro D. Crispino J. D. Hijiya N. Giles F.. (2015). Targeting novel signaling pathways for resistant acute myeloid leukemia. Mol. Genet. Metab. 114, 397–402. 10.1016/j.ymgme.2014.11.017 25533111 PMC4355162

[B22] Tibshirani R. Hastie T. Narasimhan B. Chu G. (2002). Diagnosis of multiple cancer types by Shrunken centroids of gene expression. Proc. Natl. Acad. Sci. U.S.A. 99, 6567–6572. 10.1073/pnas.082099299 12011421 PMC124443

[B23] Vaske C. J. Benz S. C. Sanborn J. Z. Earl D. Szeto C. Zhu J.. (2010). Inference of patient-specific pathway activities from multi-dimensional cancer genomics data using PARADIGM. Bioinformatics 26, i237–i245. 10.1093/bioinformatics/btq182 20529912 PMC2881367

[B24] White R. T. Damm D. Hancock N. Rosen B. Lowell B. Usher P.. (1992). Human adipsin is identical to complement factor d and is expressed at high levels in adipose tissue. J. Biol. Chem. 267, 9210–9213. 1374388

[B25] Xu Y. Ding Y. Li X. Wu X. (2015). Cystatin c is a disease-associated protein subject to multiple regulation. Immunol. Cell Biol. 93, 442–451. 10.1038/icb.2014.121 25643616 PMC7165929

[B26] Yang A.-J. Song X.-Y. (2010). Bayesian variable selection for disease classification using gene expression data. Bioinformatics 26, 215–222. 10.1093/bioinformatics/btp638 19933163

[B27] Zhang T. (2011). Adaptive forward-backward greedy algorithm for learning sparse representations. IEEE Trans. Inform. Theory 57, 4689–4708. 10.1109/TIT.2011.2146690

[B28] Zhu J. Hastie T. (2004). Classification of gene microarrays by penalized logistic regression. Biostatistics 5, 427–443. 10.1093/biostatistics/kxg046 15208204

[B29] Zou H. Hastie T. (2005). Regularization and variable selection via the elastic net. J. R. Stat. Soc. Ser. B 67, 301–320. 10.1111/j.1467-9868.2005.00503.x

