# Supplementary material for: A Predictive Based Regression Algorithm for Gene Network Selection
Source: Front Genet. 2016 Jun 15;7:97. doi: 10.3389/fgene.2016.00097 (PMC4908120; doi:10.3389/fgene.2016.00097)
Supplement: Supplementary file 1 [file Presentation1.pdf]

Appendix: A Predictive Based Regression Algorithm for Gene Network Selection

Stéphane Guerrier[1 ⋆], Nabil Mili[2 ⋆], Roberto Molinari[2], Samuel Orso[2], Marco Avella-Medina[2] & Yanyuan Ma[3]

[1]Department of Statistics
University of Illinois at Urbana-Champaign, USA
Email: stephane@illinois.edu

[2]Research Center for Statistics
Geneva School of Economics and Management
University of Geneva, Switzerland

[3]Department of Statistics
University of South Carolina, USA

A Adapting the algorithm to p

In this subsection we provide two variants of the algorithm proposed in Section 3 in order to adapt it to situations where p is either small or large.

A.1 Adapting the algorithm to very large p

In situations where p is extremely large and the initial step of the algorithm is not computationally feasible, this step can, for example, be replaced by the following modified initial step:

A′. *Large p Modified Initial Step:* We start by augmenting our initial variable set $\mathcal{M}_0$ with $d = 1$ variable in order to construct the set $\mathcal{I}_1^*$.

1. Augment $\mathcal{M}_0$ with $d = 1$ variable selected uniformly at random in $\mathcal{J}_f$.
2. Construct B models of dimension 1 by repeating Step A′.1 B times.

3. From Steps A$'$.1 and A$'$.2, construct the set $\mathcal{I}_1^*$ using (3). Go to Step B and let $d = 2$.

A.2 Adapting the algorithm to small p

On the other hand, when p is of reasonable size it may be possible to compute and evaluate all the $\binom{p}{d'}$ models of dimension $2 \leq d' \leq d_{\max}$. In such cases, it may be feasible to also modify the initial step of the proposed algorithm to a different modified initial step. A possible modification is the following:

A$''$. *Small p Modified Initial Step:* We start by augmenting our initial varable set $\mathcal{M}_0$ with d $(1 \leq d \leq d')$ variables in order to construct the sets $\mathcal{I}_1^*, ..., \mathcal{I}_{d'}^*$.

1. We augment our initial variable set $\mathcal{M}_0$ with 1 variable in order to construct the set $\mathcal{I}_1^*$.
   (i) Construct the p possible models obtained by augmenting $\mathcal{M}_0$ with each of the p available variables.
   (ii) Compute $\widehat{D}(\cdot, \cdot)$ for every model obtained in Step (i).
   (iii) From Steps (i) and (ii), construct the set $\mathcal{I}_1^*$ using (3). Go to Step A$''$.2 and let $d = 2$.
2. We augment our initial model $\mathcal{M}_0$ set by d variables in order to construct the set $\mathcal{I}_d^*$.
   (i) Construct the $\binom{p}{d}$ possible models and augment $\mathcal{M}_0$ with all variables of these constructed models.
   (ii) Compute $\widehat{D}$ for every model obtained in Step (i).
   (iii) From Steps (i) and (ii), construct the set $\mathcal{I}_d^*$ using (3) and let $d = d + 1$. Go to Step A$''$.2 (if $d < d'$) or Step B.1 (if $d \geq d'$), with model dimension starting value d.

B Complementary results on Acute Leukemia

Table 3 reports the main biomarker hubs and related biomarker networks for the *leukemia* data set analysed in Section 4.1.

Table 4 reports the performances of our implementation of the competing methods as described in Section 5. Unlike reported in Table 1, here the proposed

	Affy ID	Gene ID	Gene Function	Biological Process
**NETWORK 1**				
Position 1	M27891_at	ENSG00000101439	Cystatin C	AA
Position 2	D80006_at	ENSG00000114978	MOB kinase activator 1A	AA
	M20778_s_at	ENSG00000163359	Collagen, type VI, alpha 3	AA
	U57316_at	ENSG00000108773	K(lysine) acetyltransferase 2A	TF
	U90549_at	ENSG00000182952	High mobility group nucleosomal binding domain 4	TF
	X66899_at	ENSG00000182944	Ewing Sarcoma region 1; RNA binding protein	TF
	M74088_s_at	ENSG00000134982	Adenomatous polyposis coli, DP2, DP3, PPP1R46	TF
	U51166_at	ENSG00000139372	thymine-DNA glycosylase	TF
	Z69881_at	ENSG00000074370	ATPase, Ca++ transporting, ubiquitous	IPT
	U49248_at	ENSG00000023839	ATP-binding cassette, sub-family C (CFTR/MRP), member 2	IPT
	X89109_s_at	ENSG00000102879	Coronin, actin binding protein, 1A	IPT
	HG2815-HT2931_at	ENSG00000092841	Myosin, Light Chain, Alkali, Smooth Muscle (Gb:U02629)	ACC
	M94345_at	ENSG00000042493	Capping protein (actin filament), gelsolin-like	ACC
	L33075_at	ENSG00000140575	IQ motif containing GTPase activating protein 1	ACC
	L07633_at	ENSG00000092010	Proteasome (prosome, macropain) activator subunit 1 (PA28 alpha)	APC
	J03589_at	ENSG00000102178	Ubiquitin-like 4A	APC
	D83920_at	ENSG00000085265	FCN1, Ficolin-1	IR
	X03934_at	ENSG00000167286	CD3d molecule, delta (CD3-TCR complex)	IR
**NETWORK 2**				
Position 1	X95735_at	ENSG00000159840	Zyxin	ACC
Position 2	X04526_at	ENSG00000185838	Guanine nucleotide binding protein (G protein), beta polypeptide 1	ST
	D78577_s_at	ENSG00000128245	Tyrosine 3-monooxygenase/tryptophan 5-monooxygenase activation protein, eta	ST
	U32645_at	ENSG00000102034	E74-like factor 4 (ets domain transcription factor)	TF
	U93867_at	ENSG00000186141	Polymerase (RNA) III (DNA directed) polypeptide C (62kD)	TF
	U29175_at	ENSG00000127616	SWI/SNF related, matrix associated, actin dependent regulator of chromatin, subfamily A, member 4	TF
	Y00291_at	ENSG00000077092	Retinoic acid receptor, beta	TF
	D17532_at	ENSG00000110367	DEAD (Asp-Glu-Ala-Asp) Box Helicase 6	TF
	HG3521-HT3715_at	ENSG00000127314	Ras-Related Protein Rap1b	TF
	M83233_at	ENSG00000140262	Transcription factor 12	TF
	U94855_at	ENSG00000175390	Eukaryotic translation initiation factor 3, subunit F	TF
	L07758_at	ENSG00000136045	PWP1 homolog	TF
	D63506_at	ENSG00000116266	Syntaxin binding protein 3	IR
	M33680_at	ENSG00000110651	CD81 molecule	IR
	HG1612-HT1612_at	ENSG00000175130	Macmarcks	CG
	M92287_at	ENSG00000112576	Cyclin D3	CG
	M60483_rna1_s_at	ENSG00000113575	Protein Phosphatase 2 (formerly 2A), catalytic subunit, alpha isoform	CG
	U84388_at	ENSG00000169372	CASP2 and RIPK1 domain containing adaptor with death domain	AA
	S80437_s_at	ENSG00000169710	Fatty acid synthase	
**NETWORK 3**				
Position 1	M84526_at	ENSG00000197766	Complement factor D (adipsin)	IR
Position 2	M28130_rna1_s_at	ENSG00000169429	Interleukine-8	IR
	Z32765_at	ENSG00000135218	CD36 - Thrombospondin receptor	IR

TABLE 3: *Biomarker network organisation -* leukemia *data set - Lymphoblastic / Myeloblastic leukemia. TF = Transcription/translation factor activity, DNA repair and catabolism - AA = apoptotic activity - IR = immunity, inflammatory response (blood coagulation, antigen presentation and complement activation) - IPT = intracellular protein trafficking, transmembrane transport - ACC = actin activity, cytoskeleton organisation - APC = protein catabolism - ST = intracelular signal transduction - CG = cell growth, proliferation and division. Source:* www.ensembl.org ; www.uniprot.org

method uses the classical tenfold-CV for $D(\cdot,\cdot)$ ($K = 1$). The other hyper-parameters are kept the same (i.e. $\alpha = 0.01$, $B = 20'000$ and $\pi = 0.5$).

**Method**	**Tenfold CV error**	**Test error**	**Number of genes**
Support vector machine (with recursive feature elimination)	0/38	5/34	2/7129
Penalised logistic regression (with forward selection followed by backward deletion)	3/38	4/34	3/7129
Logistic regression (with greedy forward selection)	1/38	3/34	1/7129
Nearest shrunken centroids	3/38	1/34	372/7129
Elastic net	0/38	2/34	74/7129
Panning Algorithm (81)			
Model a	0/38	1/34	2/7129
Model b	0/38	2/34	2/7129
Model c	0/38	2/34	2/7129
[. . .]			2/7129
Model averaging		6/34	2/7129
Trimmed-mean model averaging		2/34	2/7129

TABLE 4: *Performances of our implementation of the competing methods on the leukemia data-set. For the Panning Algorithm, models "a" to "c" are three examples out of the 81 models. All the 81 models have a tenfold-CV error of 0 except one. The best test error is 1 and the worst is 21. Model averaging gives an equal weight to all the 274 models and aggregates their prediction. Trimmed-mean model averaging is Model averaging on the best 25% models based on their in-sample deviances.*

C Breast Cancer

The second data-set we analyzed is the *breast cancer* data presented in Chin *et al.* (2006). The main goal behind analyzing this data is to identify the estrogen receptor expression on tumor cells which is a crucial step for the correct management of breast cancer. Similarly to Table 4 in Appendix B, Table 5 reports the performances of our implementation of the competing methods and the proposed approach on the *breast cancer* data. For the sake of this comparison, the data-set was randomly split into training (60) and test (58) sets. The hyper-parameters

of the proposed method are $\alpha = 0.01$, $B = 30'000$, $\pi = 0.5$ and $D(\cdot, \cdot)$ is the repeated tenfold-CV ($K = 10$).

**Method**	**Tenfold CV error**	**Test error**	**Number of genes**
Support vector machine (with recursive feature elimination)	0/60	10/58	3/22215
Penalised logistic regression (with forward selection followed by backward deletion)	2/60	12/58	15/22215
Logistic regression (with greedy forward selection)	2/60	11/58	2/22215
Nearest shrunken centroids	2/60	11/58	5/22215
Elastic net	3/60	11/58	196/22215
Panning Algorithm (274)			
Model a	0/60	9/58	3/22215
Model b	2/60	9/58	3/22215
Model c	0/60	12/58	3/22215
[...]			3/22215
Model averaging		10/58	3/22215

TABLE 5: *Performances of our implementation of the methods on the breast cancer data-set. For the proposed method, models "a" to "c" are two examples out of 274 models. The tenfold-CV error varies between 0 and 3. The best test error is 9 and the worst is 31. Model averaging gives an equal weight to all the 274 models and aggregates their prediction.*

Figure 3 shows the paradigmatic network identified by our method for the *breast cancer* data for which the selected model dimension is three (i.e. only three biomarkers are needed in a model to well classify the breast cancer). We used the hyper-parameters $\alpha = 0.01$, $B = 22'215$, $\pi = 0.05$ and for $D(\cdot, \cdot)$ the tenfold-CV repeated $K = 10$ times was used. Table 6 provides the details of the networks based on the three main hubs and is to be interpreted as described in Section 4.1.

This figure is a clear example of the advantages of the proposed method since, it not only selects a set of low-dimensional models with a high predictive power, but also provides the basis for a more general biological interpretation which takes into account interactions between different biomarkers as opposed to one single model. The three main hubs identified through the proposed algorithm are:

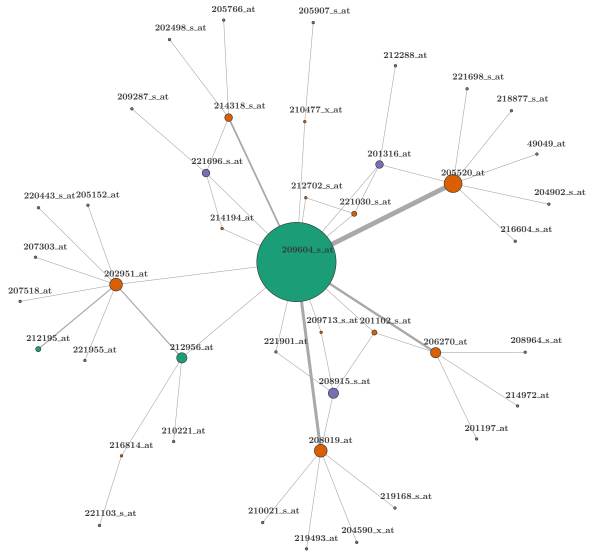

FIGURE 3: *Network representation of biomarkers selected from breast cancer data-set. Colors represent the position of covariates within the model: green for first position (hub), orange for second and purple for third. The width of the connecting lines is proportional to the frequency with which two biomarkers appear in the same model. The size of the circles is proportional to the frequency with which a biomarker is present within the selected set of models. (Note: biomarker "209602_s_at" is merged with biomarker "209604_s_at").*

1. GATA binding protein 3 (GATA3): a transcription factor regulating the differentiation of breast luminal epithelial cells;

2. IL6 Signal Transducer (IL6 ST): a pro-inflammatory cytokine signal transducer;

3. TBC1 domain family, member 9 (TBC1D9): a GTPase-activating protein for Rab family protein involved in the expression of the ER in breast tumors.

GATA3 is known to regulate the differentiation of epithelial cells in mammary glands (see Kouros-Mehr *et al.*, 2006) and is required for luminal epithelial cell differentiation. Its expression is progressively lost during luminal breast cancer progression as cancer cells acquire a stem cell-like phenotype (see Chou *et al.*, 2010). IL6 ST has been linked to breast cancer epithelial-mesenchymal transition and cancer stem cell traits (see Chung *et al.*, 2014), cancer-promoting microenvironment (see Bohrer *et al.*, 2014) and resistance (see Christer *et al.*, 2013). Moreover, this result supports the assertion by Taniguchi and Karin (2014) that IL6 ST and related cytokines are the critical lynchpins between inflammation and cancer. Finally, concerning the third biomarker, a recent publication by Andres and Wittliff (2012) has shown that the expression of the ER on the surface of breast tumor cells is highly correlated with the coordinate expression of different genes among which we can find TBC1D9 and GATA3. These two genes are not only considered as relevant genes according to the proposed method but as actual hubs of the "best" models which define the structure of the identified network. Instead of selecting a single model with many biomarkers whose interactions may be difficult to interpret, the proposed method selects a set of models with few biomarkers that allow them to be individually easy to interpret without losing the possibility of interpreting them within the larger network. This is what this paper intends with the expression "paradigmatic network" since by taking this approach it is possible to identify a set of biomarker families within which each biomarker is interchangeable with the others.

	Affy ID	Gene ID	Gene Function	Biological Process
**NETWORK 1**				
Position 1	209604_s_at	ENSG00000107485	GATA binding protein 3	TF
Position 2	205520_at	ENSG00000115808	Striatin, calmodulin binding protein	ER
Position 3	204902_s_at	ENSG00000168397	Autophagy related 4B, cysteine peptidase (APG4B, AUTL1, DKFZp586D1822, KIAA0943)	APC
	221698_s_at	ENSG00000172243	C-type lectin domain family 7, member A	IR
	49049_at	ENSG00000178498	Deltex 3, E3 ubiquitin ligase	APC
	209602_s_at	ENSG00000107485	GATA binding protein 3	TF
	216604_s_at	ENSG00000003989	Solute carrier family 7 (cationic amino acid transporter, y+ system), member 2	IPT
	218877_s_at	ENSG00000066651	TRNA methyltransferase 11 homolog	TF
	201316_at	ENSG00000106588	Proteasome (prosome, macropain) subunit, alpha type, 2	APC
Position 2	208019_at	ENSG00000147117	Zinc finger protein 157	TF
Position 3	219168_s_at	ENSG00000186654	PRR5 (Proline rich 5 (renal))	CG
	219493_at	ENSG00000171241	SHC SH2-domain binding protein 1	CG
	204590_x_at	ENSG00000139719	Vacuolar protein sorting 33 homolog A	APC
	210021_s_at	ENSG00000152669	Cyclin O	CG
	208915_s_at	ENSG00000103365	Golgi-associated, gamma adaptin ear containing, ARF binding protein 2	IPT
Position 2	214318_s_at	ENSG00000073910	Furry homolog	ACC
Position 3	205766_at	ENSG00000173991	Titin-cap (Telethonin)	ACC
	221696_s_at	ENSG00000060140	Serine/threonine/tyrosine kinase 1	CG
	202498_s_at	ENSG00000059804	Solute carrier family 2 (facilitated glucose transporter), member 3	STM
Position 2	201102_s_at	ENSG00000141959	Phosphofructokinase, liver	STM
Position 3	208915_s_at	ENSG00000103365	Golgi-associated, gamma adaptin ear containing, ARF binding protein 2	IPT
Position 2	201316_at	ENSG00000106588	Proteasome (prosome, macropain) subunit, alpha type, 2	APC
Position 3	212288_at	ENSG00000187239	Formin binding protein 1	ACC
Position 2	209713_s_at	ENSG00000116704	Solute carrier family 35 (UDP-GlcA/UDP-GalNAc transporter), member D1	STM
Position 3	208915 _s_at	ENSG00000103365	Golgi-associated, gamma adaptin ear containing, ARF binding protein 2	IPT
Position 2	212702_s_at	ENSG00000185963	Bicaudal D homolog 2	ACC
Position 3	221030_s_at	ENSG00000138639	Rho GTPase activating protein 24	ACC
Position 2	212956_at	ENSG00000109436	TBC1 domain family, member 9 (with GRAM domain)	IPT
Position 3	210221_at	ENSG00000080644	Cholinergic receptor, nicotinic, alpha 3 (neuronal)	ITT
Position 2	214194_at	ENSG00000083520	DIS3 mitotic control homolog (Ribosomal RNA-processing protein 44)	TF
Position 3	221696_s_at	ENSG00000060140	Serine/threonine/tyrosine kinase 1	CG
Position 2	216814_at	ENSG00000232267	ACTR3 pseudogene 2	PUP
Position 3	221103_s_at	ENSG00000206530	Cilia and flagella associated protein 44	ACC
Position 2	221030_s_at	ENSG00000138639	Rho GTPase activating protein 24	ACC
Position 3	201316_at	ENSG00000106588	Proteasome (prosome, macropain) subunit, alpha type, 2	APC
Position 2	221696_s_at	ENSG00000060140	Serine/threonine/tyrosine kinase 1	CG
Position 3	209287_s_at	ENSG00000070831	Cell division control protein 42 homolog	ACC
Position 2	221901_at	ENSG00000138944	KIAA1644	PUP
Position 3	208915_s_at	ENSG00000103365	Golgi-associated, gamma adaptin ear containing, ARF binding protein 2	IPT
Position 1	209602_s_at	ENSG00000107485	GATA3	TF
Position 2	202951_at	ENSG00000112079	Serine/threonine kinase 38	CG
Position 3	220443_s_at	ENSG00000116035	VAX2 (ventral anterior homeobox 2)	TF
	221955_at	ENSG00000088256	Guanine nucleotide binding protein (G protein), alpha 11 (Gq class)	ITT
	207303_at	ENSG00000154678	Phosphodiesterase 1C, calmodulin-dependent 70kDa	ST

	205152_at	ENSG00000157103	Solute carrier family 6, member 1	ST
	207518_at	ENSG00000153933	Diacylglycerol kinase, epsilon 64kDa	ST
Position 2	206270_at	ENSG00000126583	Protein kinase C, gamma	ST
Position 3	208964_s_at	ENSG00000149485	Fatty acid desaturase 1	FAM
	201197_at	ENSG00000123505	Adenosylmethionine decarboxylase 1	CG
	201102_s_at	ENSG00000141959	ATP-dependent 6-phosphofructokinase, liver type	STM
	214972_at	ENSG00000198408	Protein O-GlcNAcase (Meningioma expressed antigen 5 (hyaluronidase))	ST
Position 2	210477_x_at	ENSG00000107643	Mitogen-activated protein kinase 8	CG
Position 3	205907_s_at	ENSG00000127083	Osteomodulin	STM
**NETWORK 2**				
Position 1	212195_at	ENSG00000134352	IL6 Signal Transducer	ICT
Position 2	202951_at	ENSG00000112079	Serine/threonine kinase 38	CG
Position 3	221955_at	ENSG00000088256	Guanine nucleotide binding protein (G protein), alpha 11 (Gq class)	ITT
	207303_at	ENSG00000154678	Phosphodiesterase 1C, calmodulin-dependent 70kDa	ICT
**NETWORK 3**				
Position 1	212956_at	ENSG00000109436	TBC1 domain family, member 9 (with GRAM domain)	IPT
Position 2	202951_at	ENSG00000112079	Serine/threonine kinase 38	CG
Position 3	205152_at	ENSG00000157103	Solute carrier family 6, member 1	ST
	207518_at	ENSG00000153933	Diacylglycerol kinase, epsilon 64kDa	ST
Position 2	216814_at	ENSG00000232267	ACTR3 pseudogene 2	PUP
Position 3	221103_s_at	ENSG00000206530	Cilia and flagella associated protein 44	ACC

TABLE 6: *Biomarker network organisation* - breast cancer *data set - Estrogen Receptor - Breast Cancer.*
TF = Transcription/translation factor activity, DNA/RNA repair and catabolism - ER = estrogen receptor activity - APC = autophagy - protein catabolism - IR = immunity, inflammatory response (blood coagulation, antigen presentation and complement activation) - CC = cell/cell communication - ST = intracellular signal transduction, protein glycosylation - CG = cell growth and division - IPT = intracellular protein trafficking , transmembrane amino-acid transporter - ACC = actin activity, cytoskeleton organisation, cell projection - STM = sugar transport and metabolism - ITT = ion transmembrane transport, transmembrane signaling systems - PUP = pseudogene, uncharacterized protein - FAM = fatty acid metabolism. Source: www.uniprot.org ; www.ncbi.nlm.nih.gov/gene

References

Andres, S. A. and Wittliff, J. L. (2012). Co-expression of genes with estrogen receptor-α and progesterone receptor in human breast carcinoma tissue. *Hormone molecular biology and clinical investigation*, **12**(1), 377–390.

Bohrer, L. R., Chuntova, P., Bade, L. K., Beadnell, T. C., Leon, R. P., Brady, N. J., Ryu, Y., Goldberg, J. E., Schmechel, S. C., Koopmeiners, J. S., *et al.* (2014). Activation of the fgfr–stat3 pathway in breast cancer cells induces a hyaluronan-rich microenvironment that licenses tumor formation. *Cancer research*, **74**(1), 374–386.

Chin, K., DeVries, S., Fridlyand, J., Spellman, P. T., Roydasgupta, R., Kuo, W.-L., Lapuk, A., Neve, R. M., Qian, Z., Ryder, T., *et al.* (2006). Genomic and transcriptional aberrations linked to breast cancer pathophysiologies. *Cancer cell*, **10**(6), 529–541.

Chou, J., Provot, S., and Werb, Z. (2010). Gata3 in development and cancer differentiation: cells gata have it! *Journal of cellular physiology*, **222**(1), 42–49.

Christer, H., Peter, K., Margaret, L. A., Stephen, H., and Kathryn, M. T. (2013). A mechanism for epithelial-mesenchymal transition and anoikis resistance in breast cancer triggered by zinc channel zip6 and stat3 (signal transducer and activator of transcription 3). *Biochemical Journal*, **455**(2), 229–237.

Chung, S. S., Giehl, N., Wu, Y., and Vadgama, J. V. (2014). Stat3 activation in her2-overexpressing breast cancer promotes epithelial-mesenchymal transition and cancer stem cell traits. *International journal of oncology*, **44**(2), 403–411.

Kouros-Mehr, H., Slorach, E. M., Sternlicht, M. D., and Werb, Z. (2006). Gata-3 maintains the differentiation of the luminal cell fate in the mammary gland. *Cell*, **127**(5), 1041–1055.

Taniguchi, K. and Karin, M. (2014). Il-6 and related cytokines as the critical lynchpins between inflammation and cancer. In *Seminars in immunology*, volume 26, pages 54–74. Elsevier.
